# On the Dry Dressing Treatment of Wounds

**Published:** 1883-11-20

**Authors:** Samson Gamgee


### ON THE "DRY DRESSING" TREATMENT OF WOUNDS.

By SAMSON GAMGEE, M. D.

Generalizations are proverbially difficult in a science and practice like that of surgery. However sound be their foundations, however close the reasonings by which they are arrived at, their success in particular cases depends on the judgment, skill and care with which they are applied. To the reservations already made I must add something on "Dry Dressing," which, unqualified, is a very misleading designation of this plan of treatment. It is certainly entitled to be called "dry dressing," inasmuch as water is not used, and even astringent and antiputrescent lotions very sparingly so; but success demands attention to all essentials of the physiological treatment of surgical injuries—immobility, position, and pressure, drainage and infrequent dressing, pure and non-putrescent materials; gentle, patient, and skilled manipulation; intelligent and unceasing watchfulness of constitutional states.

Fresh wounds without loss of substance are particularly suited for the plan of treatment here recommended. They should be put up without water, the edges accurately in contact; always bearing in mind the necessity of providing for drainage outward of any effused fluid. Under absorbent pads and elastic pressure, with absolute rest and attention to position, the vast majority of fresh wounds heal rapidly, solidly, and painlessly. When the dressing is changed, which it should only be infrequently, no water should be employed; but if there be any discharge and necessity for cleaning, this can best be done with a pledget of dry lint or of absorbent gauze and cotton; all manipulations to be of the lightest. Such dry dressing simulates the natural scabbing process, but is really more perfect. Wounds of many inches in length heal so directly and perfectly under dry dressing and elastic pressure, that in the course of a few days it is often difficult to detect the fine linear scar on the dry and shrivelled skin. If a fresh wound be attended with loss of substance, some boroglycerine should be poured on the part before application; it prevents too close adhesiveness, and possible bleeding, when the dressing is removed, and has the further advantage of preventing decomposition.

The necessary employment of sutures and adhesive plasters, according to requirements, need not be dwelt upon, and I shall only briefly remark that instead of, or in addition to, such bonds of union, I frequently employ styptic colloid, compound tincture of benzoin or collodion.

In wounds with large loss of substance, if healing be slow, action may profitably be stimulated by a variety of the well known astringent applications in ointment or lotion, than which I do not know a better than the old red lotion, with a liberal addition of glycerine. Position, rest, and pressure remain cardinal indications, poultices and water prohibited. By this I mean stagnant water in the shape of water dressing, which is nearly as potent as a poultice in promoting suppuration and decomposition. It is otherwise with cold water irrigation, which is consistent with, nay may be made conducive to, perfect drainage, and by its astringent and sedative action produces effects very similar to those of rest and pressure. Cold irrigation is not easy to apply continuously comfortably, and one of its great advantages, the low temperature, may be secured by ice bags.

I hope I have made it clear that while the absence of water is a prominent feature of the "Dry Dressing" method, an essential is the maintenance of immovable apposition under elastic pressure, whereby the dynamics of the circulation are so controlled that the part is only allowed blood enough to nourish it. Irrigation, the great cause of stasis and effusion, is reduced to a minimum, and the part is maintained in a state the nearest approaching to inaction and dryness. In direct proportion the material and the possibilities of decomposition are averted.

Contused and inflamed wounds likewise afford conclusive evidence of the soundness and general applicability of the principles and method just related. The dressing which I hold in my hand was removed from one of the employees in an iron warehouse. He was moving some pigs of iron, when one, weighing a little over a hundred weight, fell on his right foot. I saw the case very shortly afterward, and found the foot very much swollen, its bony outline obliterated, the skin bluish and shining, with a star-shaped wound on the center of the instep. Having satisfied myself that no foreign body was present, I dried the wound and placed over the dorsum of the foot this large pad of absorbent gauze and cotton, and then a compressive bandage from the roots of the toes to the middle of the leg. I enjoined my patient to keep perfectly quiet, lying during the day with his head at the foot of a sofa and the injured foot over its head. I did not remove the dressing until the eighth day, when the wound was healed, the outline of the limb perfect, and though the skin was mottled, as from a bruise, up to the middle of the leg, it was cool and painless.

You see how the blood had penetrated, though in small quantity, through the dressings, and dried on the outside. The tincture of benzoin had acted as a coagulant and anti-putrescent, and drying into the lint served the purposes of a mould. Its styptic property was assisted by pressure and position, under which the effusion was absorbed; the part shrank, and the wound healed without further interference. This result, a typical one of the method, was not a simple consequence of a dry application, but due to a variety of causes which combined in controlling the circulation and promoting reparative action in accordance with demonstrably true principles of animal physics.—*Lancet.*

